# The Interface between Nanoenergy and Self-Powered Electronics

**DOI:** 10.3390/s21051614

**Published:** 2021-02-25

**Authors:** Yi-Lin Wang, Hai-Tao Deng, Zhen-Yu Ren, Xin-Tian Liu, Yu Chen, Cheng Tu, Jun-Lian Chen, Xiao-Sheng Zhang

**Affiliations:** School of Electronic Science and Engineering, University of Electronic Science and Technology of China, Chengdu 611731, China; wangyilin@std.uestc.edu.cn (Y.-L.W.); htdeng@std.uestc.edu.cn (H.-T.D.); rjiaryfie@gmail.com (Z.-Y.R.); xintian.liu98@gmail.com (X.-T.L.); ctu@uestc.edu.cn (C.T.)

**Keywords:** nanogenerator, triboelectric, self-powered, nanoenergy

## Abstract

In recent decades, nanogenerators based on several techniques such as triboelectric effects, piezoelectric effects, or other mechanisms have experienced great developments. The nanoenergy generated by nanogenerators is supposed to be used to overcome the problem of energy supply problems for portable electronics and to be applied to self-powered microsystems including sensors, actuators, integrated circuits, power sources, and so on. Researchers made many attempts to achieve a good solution and have performed many explorations. Massive efforts have been devoted to developing self-powered electronics, such as self-powered communication devices, self-powered human–machine interfaces, and self-powered sensors. To take full advantage of nanoenergy, we need to review the existing applications, look for similarities and differences, and then explore the ways of achieving various self-powered systems with better performance. In this review, the methods of applying nanogenerators in specific circumstances are studied. The applications of nanogenerators are classified into two categories, direct utilization and indirect utilization, according to whether a treatment process is needed. We expect to offer a line of thought for future research on self-powered electronics.

## 1. Introduction

Innovations in micro-/nano-fabrication technology and advanced materials have provided positive conditions for updating electronic devices [1,2,3]. Nowadays, electronics are being developed towards the goals of miniaturization, multi-functionality, high levels of integration, and light weight [4,5,6,7]. Portable electronics have penetrated various aspects of our daily lives and raise our quality of life. Many technologies that are supposed to change our future, such as the Internet of Things, human–machine interfaces, and artificial intelligence, all benefit from electronic innovation [8,9,10,11]. Among these, many specific applications are discrete devices, so they need be powered separately. Most of the electronic devices are portable and are carried by humans. At present, the main way to supply power is by equipping the device with a battery. As a result, huge and heavy batteries must be carried, which, combined with the problems of limiting use time and frequent charging, hinder the portability and sustainability of wearable electronics.

To solve the problem of unsustainable power supplies, researchers intend to innovate batteries [12,13] or to find another power source as a substitute. One way to substitute for a battery is by harvesting energy from the living environment [14,15,16,17,18], where plenty of energy exists in various forms, such as solar radiation, temperature gradients, and mechanical movement. Recently, several power generation mechanisms that can convert ambient energy into electronic energy, such as through the triboelectric effect, the piezoelectric effect, the thermoelectric effect, the photoelectric effect, or electromagnetic induction, have been developed for portable electronics, and these allow portable electronics to be self-powered. Many types of nanogenerators have also been proposed, including triboelectric nanogenerators (TENGs) [19,20], piezoelectric nanogenerators (PENGs) [21,22], thermoelectric generators (TEGs) [23], and so on. Considering that a nanogenerator is realized by nanomaterials and nanotechnology, on the other hand, the energy generated by a nanogenerator is used for micro/nanosystems [24], so we describe the nanogenerator’s output as nanoenergy. 

Previous works have reported many different types of nanogenerators and have shown their application. Many of the applications focus on self-powered electronics [25,26,27]. In addition, many review articles have studied the characteristics of different nanogenerators and their introduction in many specific fields, such as biomedical monitoring [28,29], the Internet of Things [30,31], and environmental monitoring [32,33]. However, there are few studies about how the nanogenerator can be given a specific application. In this review, we explore the intermediate process between making a nanogenerator output and putting the output to use, i.e., the interface between nanoenergy and self-powered electronics, and we classify several research achievements into two categories, namely direct utilization and indirect utilization, according to whether a treatment process is needed. Specifically, direct utilization refers to systems in which the outputs of nanogenerators directly drive or control actuators or directly show information on instruments, while indirect utilization refers to systems in which there are some intermediate steps between the output of the nanogenerator and the operation of the actuator, rather than a direct delivery of the output to the actuators.

## 2. Direct Utilization

For some applications, the output of nanogenerators can be used to directly realize some functions without additional processing. We call this method of using nanoenergy direct utilization. In this method, the output is directly applied to the functional part directly or is directly analyzed by instruments. Specifically, the electrostatic field and the electrostatic force generated by the nanogenerator are used directly, or the features of the output can directly be used to show some specific physical states. In this section, four types of direct utilization are summarized according to the details of the functions of the nanoenergy.

### 2.1. Directly Reflecting States

**Figure 1:**
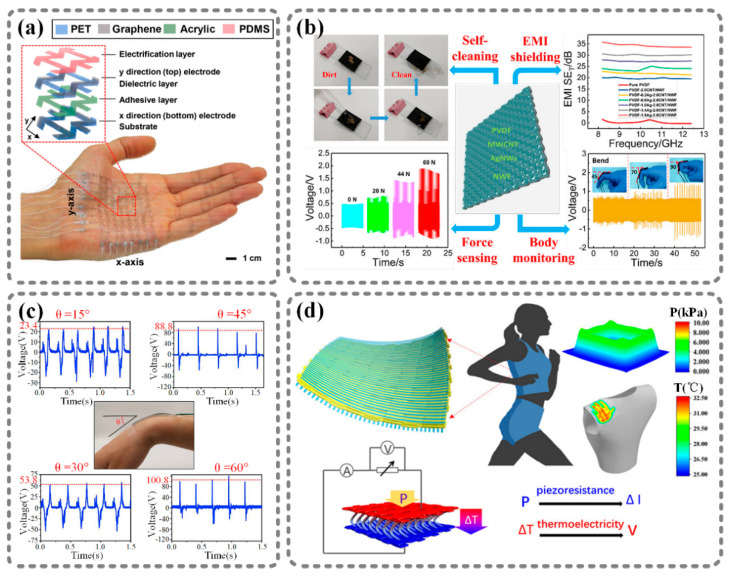
Nanogenerator-based self-powered sensors that directly reflect environmental status. (**a**) Graphene-based stretchable triboelectric nanogenerator (TENG): wearable self-powered touch sensor [44]. (**b**) As-synthesized Ag nanowires and PVDF(polyvinylidene fluoride)-based composite: PVDF-based sensor for force sensing and body monitoring [45]. (**c**) Printed silk-fibroin-based triboelectric nanogenerators: triboelectric nanogenerator (TENG) for angle sensing [46]. (**d**) Poly(3,4-ethylenedioxythiophene):poly(styrene sulfonate) (PEDOT:PSS)-based 3D thermoelectric spacer fabric: self-powered pressure-temperature sensing e-skin [47]. Reproduced with permission from Elsevier [44,46] and American Chemical Society [45,47].

The ability of nanogenerators to convert external stimuli into electricity with unique electrical characteristics provides the potential for nanogenerators to serve as self-powered sensors. Therefore, just by directly reading the output signals of nanogenerators, people or machines can detect environmental changes in real time. In recent years, the output characteristics of many different types of nanogenerators have been demonstrated to indicate motions and gestures [34,35], acceleration [36,37], force [38,39], temperature [40,41], and humidity [42,43], according to the quantitative relationship between external stimuli and the waveform and amplitude of the electrical output. In this section, four typical self-powered sensors are selected, and they are summarized in Figure 1. 

Figure 1a shows a wearable self-powered touch sensor based on graphene [44], which is as thin and stretchable as skin. The excellent conformability of the sensor enables it to be attached to human skin, to recognize the position of a touch point, and to track a touch movement without an external power supply. This sensor is an 8 × 8 single-electrode TENG array. When the external force is applied to the nanogenerator, it generates an output voltage corresponding to a pressure between 10.6 and 101.7 kPa. By monitoring and analyzing the output voltage of each unit in the array, we can recognize which unit is pressed and, thereby, detect the touch position and touch movement in real time. Recently, many works have focused on the measurement of force. For example, Sang et al. developed a PVDF-based sensor for force sensing and body monitoring [45], as shown in Figure 1b. In this work, PVDF was used as the piezoelectric material. If an external force is applied on the sensor, the PVDF will be deformed, and a voltage will be generated through an electrode composed of silver nanowires and multiwall carbon nanotubes inside the PVDF. The output voltage and the force have a linear relationship, so we can measure the force by directly reading the output voltage. In addition, different body motions lead to different deformations of the PVDF; therefore, this sensor also has a body monitoring ability. In addition, Wen et al. demonstrated that a TENG can be utilized to detect changes in angles, as shown in Figure 1c [46]. In this work, a printed silk-fibroin-based TENG was fabricated. The device was flexible and conformable, and could be attached to mobile joints. The author attached this device to a human’s wrist. Bending the wrist at different angles of 15°, 30°, 45°, and 60° induced the single-electrode TENG to generate distinctive outputs due to the different friction intensities and contact times. Based on the quantitative relationship between the output voltage and the bending angle, the nanogenerator can sense changes in the angle. Moreover, Figure 1d illustrates a thermoelectric generator-based self-powered pressure–temperature-sensing e-skin derived from a spacer fabric modified with an organic thermoelectric polymer, poly(3,4-ethylenedioxythiophene):poly(styrene sulfonate) (PEDOT:PSS) [47]. Due to the thermoelectric characteristics of PEDOT:PSS, the temperature gradient in the sheet-thickness direction of the device results in a voltage signal. In addition, the deformation of PEDOT:PSS that results from external pressure changes the conductivity of the PEDOT:PSS fibers, which will change the current output under a constant voltage, thereby converting the pressure stimulus into a current signal. By analyzing the output voltage and the change in the output current, we can obtain the temperature and pressure information, respectively.

### 2.2. Directly Driving Movable Structures

**Figure 2:**
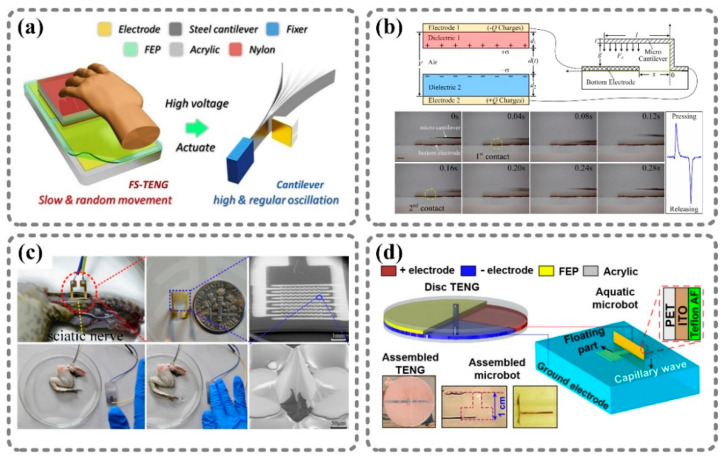
Examples of movable structures directly driven by nanogenerators. (**a**) Motion-triggered cantilever beam: self-powered cantilever system [48]. (**b**) Self-powered micro-cantilever system: micro-cantilever driven by a TENG [49]. (**c**) Self-powered nerve stimulator: stimulation of the sciatic nerve of a frog by the output of a TENG [50]. (**d**) Self-powered electrowetting-on-dielectric actuator: TENG-powered micro-bot [51]. Reproduced with permission from Elsevier [48,49,50] and American Chemical Society [51].

There are electrostatic fields and charges that exist during the operation process of a TENG. These electrostatic forces are powerful enough to directly drive or move some lightweight structures without any other power supply. According to this mechanism, nanogenerators can be used to make microstructures move in specific forms. Several works reported the use of TENGs to drive movable structures, and four typical examples are shown in Figure 2.

Figure 2a,b illustrate cantilevers driven by TENGs. Traditionally, the cantilever is driven by an alternating current (AC), and the voltage frequency must match the intrinsic resonant frequency of the cantilever [52]. As a result, cantilever systems with different parameters require drivers with varying frequencies. In contrast, these two works used TENGs as universal drivers for cantilevers with different resonant frequencies. A freestanding-mode TENG was used as a high-voltage source, as shown in Figure 2a [48]. In this work, a steel cantilever was fixed at one end, and the other end was close to a trigger electrode. The steel cantilever and the trigger electrode were connected to the two electrodes of the TENG. When the TENG was operated by hand, opposite charges would flow from the two electrodes and accumulate at the cantilever and the trigger electrode, respectively. Due to the electrostatic force, the cantilever would bend towards the trigger electrode until making contact and discharging; then, the cantilever would spring back. With the cyclical accumulation and disappearance of charges, the cantilever was able to oscillate regularly at a high frequency, which was related to the operation speed of the TENG. As for Figure 2b, the author demonstrated that the proposed contact–separation-mode TENG could successfully drive an aluminum micro-cantilever to approach the copper electrode below [49]. Herein, the cantilever was fabricated with a thickness of 11 μm by using by micro-electro-mechanical system (MEMS) technology. The cantilever and the bottom electrode were directly connected to the two electrodes of the TENG, while the air gap between the cantilever and the electrode was about 400 μm. Once the TENG was pressed or released, the cantilever would be pulled downwards to the bottom electrode by the electrostatic force. Therefore, this TENG-controlled micro-cantilever system was supposed to be used as a non-contact-mode RF MEMS switch. Both studies utilized the electrostatic field generated by TENGs, which can drive movable micro-structures to realize simple motions. Similarly, Zheng et al. developed a dual-stimulus flexible actuator by combining vapor-responsive PDMS and a TENG [53]. The actuator showed fast and controllable actuation motions under the electrostatic force from the TENG without any other power supply. 

It is worth mentioning that Zhang et al. applied a TENG to stimulate a frog’s sciatic nerve through a microneedle electrode array (MEA), as shown in Figure 2c [50]. The MEA, which consisted of 9 × 9 Si-based tips covered by a 4 μm gold layer, was implanted into frog tissue, and the tips pricked the sciatic nerve. The output of the TENG was directly applied to the electrodes of the MEA without an external circuit. Once a force was applied to the TENG, the instantaneous current flowed through the sciatic nerve via the microneedle tips. Therefore, the sciatic nerve was stimulated by the TENG’s output current and actuated the leg muscle of the frog. This study showed that a TENG’s output can not only drive mechanical structures, but can also directly cause biomedical tissues to move. There have also been many other studies on controlling biological tissues with TENGs, such as that of Lee et al., who used a TENG to directly stimulate peripheral [54], muscle [55], and pelvic nerves [56].

Figure 2d shows a TENG-powered electrowetting-on-dielectric (EWOD) micro-bot [51]. This micro-bot consisted of an EWOD actuator stuck to a floating part and a freestanding-mode disc TENG as a power source. The indium tin oxide (ITO)-coated PET layer played a major role in the EWOD actuator. The positive electrode of the disc TENG was connected to the ITO, which acted as the electrode of the EWOD actuator, while the negative electrode of the disc TENG was immersed in water as a ground electrode. The working TENG alternately generated charges on the ITO, thus changing the surface energy of the EWOD actuator and leading to capillary wave propagation. The reaction force of the capillary wave drove the micro-bot to move on the water’s surface.

### 2.3. Directly Controlling Particles

**Figure 3:**
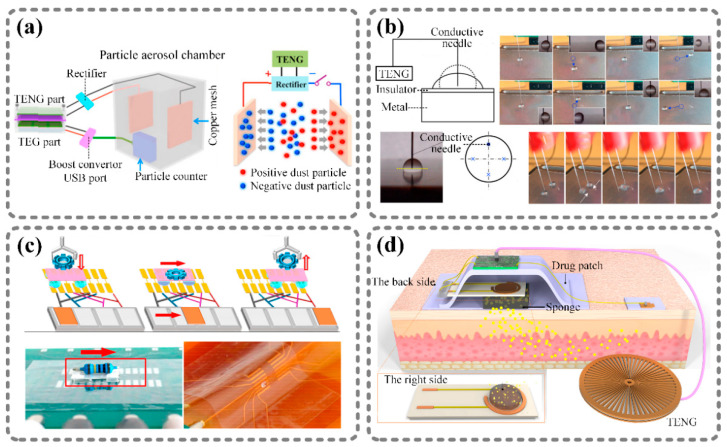
Direct control of solid particles and liquid droplets by nanogenerators. (**a**) Gas purification system based on a TENG [60]. (**b**) TENG-based liquid droplet control device: control of liquid droplets with a TENG [61]. (**c**) Self-powered transport system based on microfluids: PVDF pallet with four droplets can be manipulated to move on aluminum foils [62]. (**d**) Electric-stimulated porous polypyrrole film: self-powered drug delivery system [63]. Reproduced with permission from Elsevier [60,61,63] and American Chemical Society [62].

TENGs are known for their high voltage output, which enables them to operate like pumps in order to move particles, including solid particles and liquid particles. The particles are forced to move in a direction decided by the electric field generated by the TENG. Many efforts have focused on this mechanism and realized particle control technologies, including for air purification [57], microfluidics [58], and drug delivery [59]. Four particle control technologies are illustrated in Figure 3.

A gas purification system is shown in Figure 3a [60]. There are two copper sheets connected to the electrodes of the TENG, which offers a high voltage for electrostatic precipitation. The freely diffusing particles are usually charged positively or negatively. When the charged particles flow between metal sheets, the electric field generated by the TENG will force the positively and negatively charged particles to move towards the cathode and anode, respectively, according to Coulomb interactions. As a result, the above principle can be applied to air purification and electrostatic precipitation. Figure 3b shows an example of the control of liquid droplets with a TENG [61]. A conductive needle that was connected to a TENG’s output electrode was inserted into a droplet of NaCl solution. The droplet was placed on a Teflon tape covering a metal sheet. When the output charges of the TENG were transferred to the surface of the droplet, the opposite charge appeared on the surface of the metal sheet below the droplet due to the electrostatic induction effect. Then, the shape of the droplet changed. In addition, the droplet was able to move in different directions depending on the relative positions of the needle and droplet. In this way, the author made two droplets move towards each other, and they were eventually mixed.

Based on droplet control, some researchers realized the transportation of larger objects. For example, Nie et al. designed a self-powered microfluidic transport system based on an electrowetting technique and a TENG [62], as shown in Figure 3c. This system consisted of a freestanding-mode TENG and two rows of grating track electrodes covered with a hydrophobic layer. The track electrodes were connected to the TENG’s electrodes in the proper order. First, a droplet was placed on the hydrophobic layer. Due to the friction, the droplet was positively charged, while the track electrode just below it was negatively charged. When the freestanding layer of the TENG moved right, the right track electrode became negative and the track electrode right below the droplet became positive. Then, the droplet was driven by Coulomb forces and moved toward the right electrode. Hence, the author manipulated a mini-vehicle with this microfluidic transport system. The mini-vehicle was composed of a PVDF pallet with four droplets at the four corners, and the four droplets were on the two rows of track electrodes. By sliding the freestanding layer of the TENG, the droplets were able to control the mini-vehicle to move left or right without an external power supply.

Self-powered drug delivery is a typical application of particle control in the biomedical field. Ouyang et al. reported a self-powered transdermal drug delivery system based on a TENG, as shown in Figure 3d [63]. This drug delivery system mainly consisted of a radially arrayed TENG and a drug-loaded porous polypyrrole film. The TENG acted as a voltage stimulation, while the porous polymer film acted as a drug carrier. Polypyrrole is a kind of electricity-stimulated polymer; the drug molecules that were initially loaded into the polymer matrix were released if the proper negative voltage was applied on the polypyrrole film. When drugs were demanded, simply rotating the TENG by hand would trigger the electricity-responsive drug carrier to release drugs. In addition, the drug release rate could be controlled by adjusting the TENG operation duration.

### 2.4. Directly Modulating Electric Characteristics

In some applications, the output of a nanogenerator is utilized to modulate electric characteristics, especially the current and charges. For example, a new research field called “tribotronics” [64] focuses on controlling and tuning the transport of semiconductors by using triboelectricity. Herein, a TENG is integrated with a field-effect transistor (FET), and the output voltage of the TENG acts as the gate voltage of the FET to modulate the current between the drain and source. [65].

**Figure 4:**
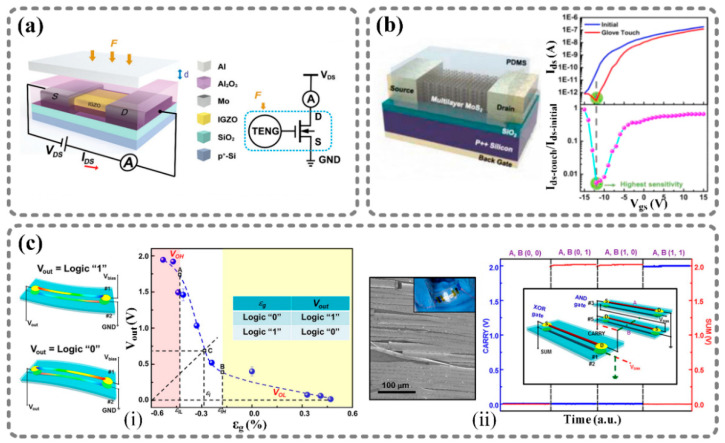
Using nanoenergy to directly modulate electronic signals and construct novel components. (**a**) InGaZnO thin-film transistor: TENG-based tribotronic thin-film transistor for tactile detection [66]. (**b**) Tribotronic MoS_2_ touch memory: zero-writing-power tribotronic touch memory [67]. (**c**) Epitaxial GaN nanobelt: logic devices based on piezoelectric transistors [68]. (i) GaN nanobelt piezotronic inverter. (ii) Piezotronic half-adder composed of GaN nanobelt piezotronic NAND and NOR logic gates. Reproduced with permission from John Wiley and Sons [66], Elsevier [67], and American Chemical Society [68].

Figure 4a shows a tribotronic thin-film transistor (TFT) developed by Cao et al. [66]. Differently from traditional TFTs, this tribotronic TFT has no gate electrode, but the source, drain, and channel are covered with Al_2_O_3_. The Al_2_O_3_ layer is combined with an Al foil to form a contact–separation-mode TENG. When an external force causes the Al foil and the Al_2_O_3_ layer to make contact and then separate, charges will be generated on the surface of the Al_2_O_3_ layer, leading to a repulsion effect on electrons in the channel, which will change the conductivity of the channel, thus modulating the TFT’s drain–source current. Moreover, this kind of tribotronic TFT was used to create a monolithic sensing array that was able to realize tactile perception. In another study, researchers used a TENG to control the threshold voltage of a two-dimensional MoS_2_ channel, and further designed a zero-writing-power touch memory technology, as shown in Figure 4b [67]. By touching the PDMS friction layer, triboelectric charges will appear on the PDMS layer and remain for about one hour. The electrostatic potential generated by triboelectric charges acts as a gate bias to modulate the electronic transport in the MoS_2_ channel. If a constant voltage is applied on the drain and source electrodes, the drain–source current will be different depending on whether the PDMS layer is touched. Thus, a touch motion can be memorized by this device without external power.

In addition to triboelectric effects, piezoelectric effects can also be used to modulate certain electronic characteristics, thereby structuring piezotronic logic devices. In 2013, Yu et al. reported GaN nanobelt strain-gated transistors [68]. The transistors consist of a polystyrene (PS) film substrate and a GaN nanobelt attached to the substrate. The two ends of the nanobelt are covered with a silver paste in order to act as source and drain electrodes. Once a compressive strain is applied on the transistor, a positive potential is induced in the GaN nanobelt due to the piezoelectric effect. Thus, the Schottky barrier height is reduced, which will increase the current in the transistor, presenting the “on” state. Alternatively, if a tensile strain is applied on the transistor, the strain-induced potential results in a decrease in the current, thus presenting the “off” state. Based on this GaN nanobelt strain-gated transistor, a GaN nanobelt inverter was designed by packaging two transistors on the top and bottom surfaces of the same substrate, as shown in Figure 4c(i). Similarly, an AND gate and an XOR gate were also designed and combined to construct a GaN nanobelt piezotronic half-adder, as shown in Figure 4c(ii).

## 3. Indirect Utilization

The output of nanogenerators is normally intermittent and irregular, so it is hard to directly power or control traditional electronics. On the one hand, for the utilization of energy that is generated, the generated power first needs to be stored, and then the power storage module outputs stable voltage to power the functional electronics. On the other hand, if the output is used as a control signal, a collection, analysis, and identification process is necessary because the machines under control cannot recognize the instructions contained in the raw output signal of a nanogenerator.

### 3.1. Powering Electronics through a Power Management Module

**Figure 5:**
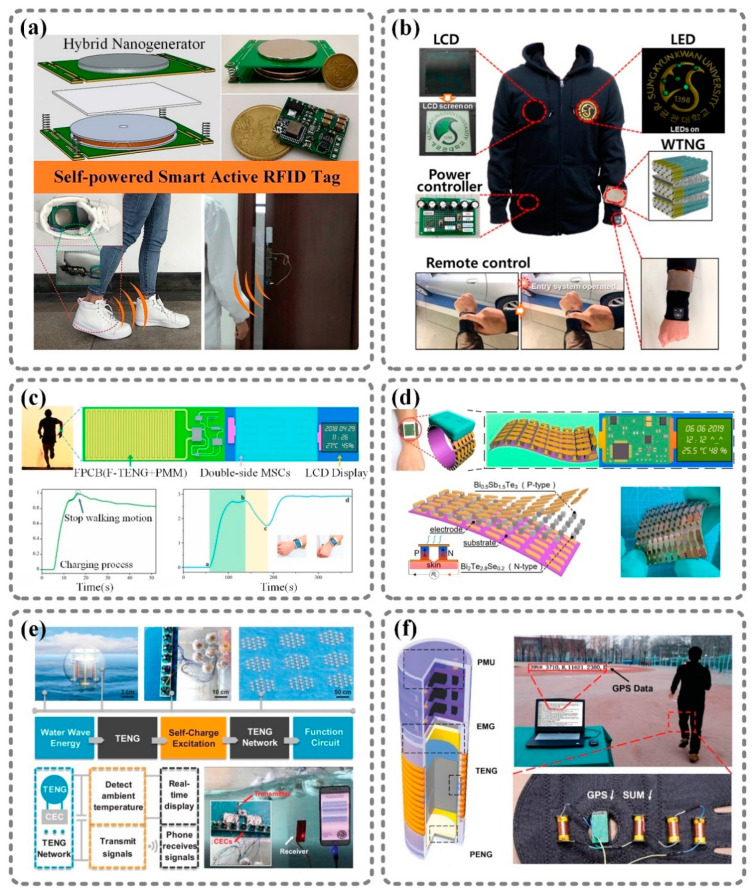
Self-powered systems with power management modules using nanoenergy. (**a**) Triboelectric-electromagnetic hybrid nanogenerator: self-powered RFID tag integrated with a hybrid nanogenerator [31]. (**b**) Nanopatterned textile-based wearable triboelectric nanogenerator: self-powered smart suit based on a textile-based wearable TENG [70]. (**c**) FPCB integrated with TENG, PMM, MSC and functional circuit: self-powered smart bracelet based on a freestanding-mode TENG [71]. (**d**) Thermoelectric grains bonding on flexible polyimide substrate: self-powered wearable monitoring system with a flexible thermoelectric generator (TEG) [72]. (**e**) TENG network with spring-assisted multilayered structure: water wave energy harvesting system that can power electronic thermometer and RF transmitter [73]. (**f**) Battery-like self-charging universal module for motional energy harvest: universal self-charging module composed of a “three-in-one” hybrid nanogenerator (electromagnetic generator (EMG), TENG, and piezoelectric nanogenerator (PENG)) and power management unit, which can support a GPS system. [74]. Reproduced with permission from John Wiley and Sons [73,74], Elsevier [31,71,72], and American Chemical Society [70].

Nanogenerators have been proven to be a potential substitute for traditional batteries in the field of portable electronics because of their sustainability, ability to harvest energy from the living environment, and potential to be integrated into systems [69]. Many researchers have tried to realize self-powered electronic systems by using nanogenerators. Here, nanogenerators are regarded as a power source, and their only requirement is to deliver power to the functional parts efficiently and stably. Usually, a power management module serves as the interface between the nanoenergy and the electronics to maximize the energy conversion efficiency, to provisionally store the pulsed energy in capacitors, and to provide stable energy for systems. Figure 5 shows six examples of self-powered electronic systems based on nanogenerators and power management modules.

Figure 5a shows a self-powered active RFID tag [31]. This RFID tag is powered by a wearable triboelectric–electromagnetic hybrid nanogenerator. This nanogenerator, which is embedded in shoes, can harvest mechanical energy while a person walks. The collected energy is stored in capacitors through a power management module, which increases the energy conversion efficiency. Then, the energy is converted into 3.3 V DC power with little ripples, which is suitable for powering a Bluetooth chip integrated with an MCU. With a stable power supply from the nanogenerator, the RFID system can work sustainably, autonomously, and omnidirectionally in a large area of up to tens of meters. For wearable electronics, textile-based devices have attracted much attention because they are comfortable to carry. As shown in Figure 5b, a “self-powered smart suit” integrated with LCD, LEDs, and remote control has been reported [70]. The smart suit is powered by a textile-based TENG. This textile-based TENG, which consists of polydimethylsiloxane (PDMS)-coated ZnO nanorod arrays on a silver-coated textile substrate and a silver-coated textile, is attached to the sleeve of the smart suit to harvest mechanical energy. Importantly, a power controller is used in the smart suit to store and distribute energy. 

In addition, the integration of nanogenerators into wearable devices has been proposed. Figure 5c,d show two self-powered smart bracelets with integrated nanogenerators. The device in Figure 5c harvests mechanical energy with a flexible freestanding TENG [71]. Meanwhile, a power management module and double-sided micro-supercapacitors (MSCs) are fabricated on a flexible printed circuit board together with the TENG in order to store energy and drive the portable electronics sustainably. This smart bracelet has been demonstrated to harvest walking energy and to steadily drive a pedometer and humidity–temperature meter. The power source of the device in Figure 5d is a flexible thermoelectric generator [72]. The thermoelectric generator is designed as part of a wristband that can be worn on the human wrist and can be attached to the skin, where continuous thermal energy can be converted into electrical energy. However, the output voltage of the thermoelectric generator is only in the tens of millivolts, which is too low to drive devices. Thus, a power management module that includes a voltage booster and a nanogenerator–voltage booster impedance-matching circuit is indispensable. Due to the use of the thermoelectric generator and power management module, this sensory system consisting of a temperature/humidity micro-sensor, a micro-accelerometer, and an LCD can fulfill signal collection, data processing, and display requirements in real time.

Previously published works have demonstrated the feasibility of harvesting wave energy from water with TENGs [75,76]. Figure 5e shows a water wave energy harvesting system [73]. The part used to realize the energy conversion in this system is a TENG network that is composed of seven spherical TENGs with a spring-assisted and multilayered structure. The TENGs in the network are linked by rigid strings, and each of them is connected to a charge excitation circuit, which increases the output performance and transforms the output from alternating current into direct current. With the constant power supply from the TENG network, an electronic thermometer can measure the temperature of the environment continuously and display the temperature value on a liquid crystal display screen in real time. Furthermore, a wireless transmitter can send an RF signal with the support of the TENG network. A mobile phone with the corresponding receiver can display the information sent by the transmitter from within 10 m. In order to satisfy the power supply demands of other electronic devices, researchers have tried to enhance the output performance of nanogenerators, and hybrid nanogenerators are a typical result. Meanwhile, the simultaneous use of multiple types of nanoenergy in one system needs to be considered. Figure 5f shows a self-charging universal module (SUM) that consists of a “three-in-one” hybrid nanogenerator and a power management unit (PMU) [74]. The hybrid nanogenerator includes an electromagnetic generator (EMG), piezoelectric nanogenerator (PENG), and triboelectric nanogenerator (TENG). The PMU is composed of three full-wave bridge rectifiers and a miniature lithium battery, and it is used to transform the alternating current generated by the hybrid nanogenerator into direct current, and store it in the miniature lithium battery. This SUM is packaged in the shape of a standard AA battery, so multiple SUMs can be used as a battery pack to provide more power. The SUM pack was demonstrated to harvest mechanical energy during motion and to support a GPS device. 

The above-mentioned self-powered systems all have an energy storage unit and a power management module. Most of the power management modules are well integrated with the device and make the power usage effective and efficient. This shows that a power management module is a key part of the utilization of nanoenergy for powering electronics.

### 3.2. Controlling Machines through Signal Regulating Process

**Figure 6:**
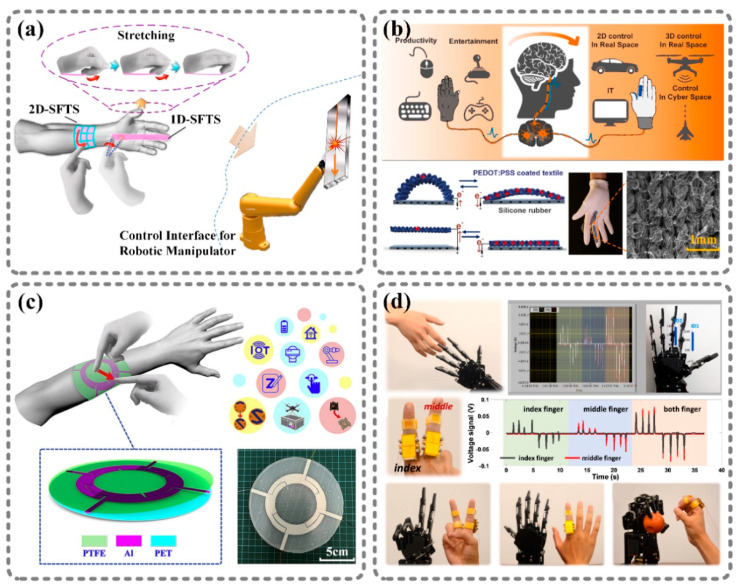
Nanoenergy is collected, analyzed, and converted into standard control signals through a signal-regulating process in order to realize human–machine interfaces. (**a**) Silicon rubber triboelectric patch: a 3D motion control interface for a robotic manipulator based on TENGs [77]. (**b**) PEDOT:PSS-coated textile: an intuitive glove-based interface for machine control. [78]. (**c**) Flexible wearable triboelectric patch: a wearable multi-functional human machine interface [79]. (**d**) Joint motion triboelectric quantization sensor: a system for the synchronous action of a human and a robotic hand [80]. Reproduced with permission from Elsevier [78,79,80] and American Chemical Society [77].

Controlling machines with motions and gestures is more intuitive and easier than doing so by typing commands. In addition, the output of a nanogenerator can reflect an operator’s motions to some extent. Thus, researchers have tried to utilize nanogenerators as human–machine interfaces, which are an important part of the Internet of Things. However, most electronics have fixed communication standards; thus, the output of nanogenerators cannot be directly applied to electronic systems as a control signal. A signal-regulating process where the nanogenerator’s output is collected, analyzed, and converted into a standard control signal is essential in order for it to serve as the interface between nanoenergy and the machine under control. Four applications of TENGs used as human–machine interfaces are illustrated in Figure 6.

Figure 6a shows a wearable, flexible 3D motion control interface [77]. The wearable interface is composed of a one-dimensional triboelectric sensor and a two-dimensional triboelectric sensor, which can generate three-dimensional information that is used to control the movement of a robotic manipulator in three-dimensional space. The output of sensors is first collected by a “signal acquisition module”; then, the signal is sent to a computer by a “filtering and amplifying module” and is processed by an analog-to-digital converter. After analyzing the information, the computer sends a command to the driver to control the robotic manipulator to move as ordered. Figure 6b shows a glove-based human–machine interface. The critical components of the device are four TENGs based on a PEDOT:PSS-coated textile [78]. These TENGs are placed on the knuckles of the glove. Bending the fingers in different ways can represent different commands. The four-channel output signals of the TENGs are collected by the MCU through the processing circuit. The MCU analyzes the signals and transmits the command to the receiver; then, the receiver acts according to the command. The glove-based human–machine interface has not only been demonstrated to control cars, drones, and other machines in real space, but also to act as a mouse or keyboard for realizing cursor control and game control in cyberspace.

A wearable control patch is shown in Figure 6c [79]. The patch consists of four aluminum electrodes that compose a splitting ring on a PET substrate and a PTFE coating. Each electrode is composed of a main middle portion and an extrusion portion, and each belongs to an independent single-electrode-mode TENG. The patch is divided into four individual areas, which correspond to the main middle portion of each electrode, and four common jointing areas, which correspond to the extrusion portions of two adjacent electrodes. When tapping or sliding operations are performed in different areas, one electrode or two adjacent electrodes will output a signal. By analyzing the output of the four electrodes with an MCU, the operation on the patch can be distinguished. Then, the operation is mapped to a specific command, and the command is transmitted to another MCU by a pair of wireless transceivers. The MCU that receives the command controls machines in order to realize the corresponding actions. 

Figure 6d shows a system for the synchronous action of a human and a robotic hand based on a triboelectric sensor [80]. The sensor, which has a hinge structure, is worn on a human finger. Due to the grating/sliding mode that is adopted in the sensor, the pulse number and the polarity of the sensor’s output relate to the flexion–extension degree, direction, and speed of the joint. A computer collects the output and analyzes the pulse pattern, maps the data to a finger motion, and then drives a robotic hand through a serial port to act according to the motion of the human’s hand, thereby realizing synchronous robot hand control.

## 4. Summary and Prospects

**Figure 7:**
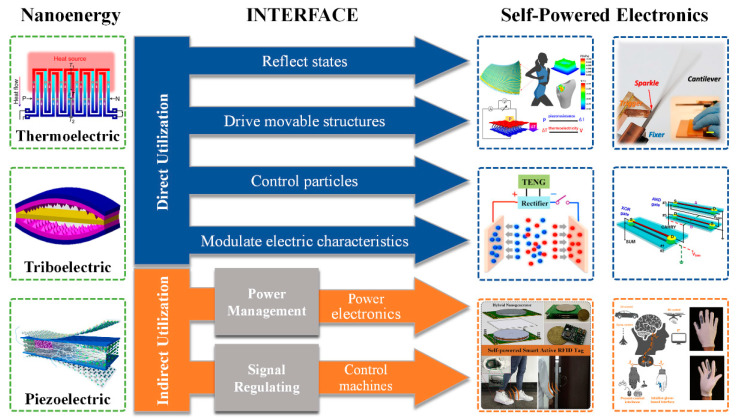
The interface between nanoenergy and self-powered electronics. Various kinds of nanoenergy, such as thermoelectric energy, triboelectric energy, and piezoelectric energy, are proposed to be used for self-powered electronics. The method of applying nanoenergy to self-powered electronics is described as the interface, which includes direct utilization and indirect utilization. For direct utilization, nanoenergy can realize active sensing, driving movable structures, controlling particles, and modulating electric characteristics. For indirect utilization, nanoenergy can power electronics and control machines with several intermediate steps such as power management modules and signal regulating process. Reproduced with permission from John Springer Nature [82], Elsevier [31,48,60,78,83], and American Chemical Society [47,68,84].

Various types of nanogenerator have been reported, and it is expected that they will be used to realize all-in-one self-powered systems [81]. However, a practical problem is presented by the use of nanoenergy generated by nanogenerators in real-world applications. The characteristics of different nanogenerators vary, so their outputs are suitable for use in different circumstances. In this review, six ways of using nanoenergy were summarized and classified into the categories of direct utilization and indirect utilization. After new nanogenerators are invented, a suitable usage can be found among these six ways according to their characteristics and practical requirements. If the output directly reflects the state of the nanogenerator and the only requirement is to know what the state is, we can directly read and analyze the output signal of the nanogenerator. If we want to control something with a nanogenerator, the methods vary. If the thing under control is a particle, a movable micro-structure, or other electrical parameters, the nanogenerator’s output can be directly applied to it. If the thing under control is a common piece of electronic equipment, it is difficult to directly drive it with nanoenergy, and a conversion process is necessary. Moreover, if the nanogenerator has a high power output and we want to use it to drive electronic systems, the energy must go through storage and treatment modules. A summary of the six ways of using nanoenergy is illustrated in Figure 7. In conclusion, the use of nanoenergy is dependent on the characteristics of the nanoenergy and the practical requirements.

Here, we studied the interface between nanoenergy and self-powered electronics in order to provide a line of thought about the practical applications of nanoenergy. Nanoenergy brings us such an attractive vision of self-powered electronics, but there are still challenges in the development process of self-powered electronics. For example, the output of self-powered sensors could be affected by other factors besides the quantity to be measured, meaning that the stability and accuracy of sensing remains to be improved. As for driving movable structures and controlling particles by nanoenergy, realizing quantitative and precise control is a common challenge. Moreover, the power of nanoenergy is not enough to support most microsystems with general power consumption and wearable electronics on the market to work continuously. In addition, there are also challenges to control machines to realize complex motion with high precision. 

However, opportunities coexist with challenges. In the future, nanoenergy is predicted to develop in the following ways. First, nanoenergy can combine with MEMS technologies to achieve high-accuracy sensing and quantitative control. Second, advanced materials and fabrication technologies will promote the output performance of nanogenerators to realize sustainable power supply. Third, nanoenergy can supply a part of the energy for ultra-low-power devices, so that it is possible to integrate nanogenerators with other ultra-low-power technologies to further reduce the device power consumption. Fourth, machine learning technologies can be adopted to process the output of nanogenerators and to realize reliable human–machine interfaces with various functions. Finally, multiple approaches summarized in the above sections can be combined to construct multi-functional smart self-powered electronic systems.

## Data Availability

Data is contained within the article.
